# Growing and cultivating the forest genomics database, TreeGenes

**DOI:** 10.1093/database/baz043

**Published:** 2019-03-13

**Authors:** Taylor Falk, Nic Herndon, Emily Grau, Sean Buehler, Peter Richter, Sumaira Zaman, Eliza M Baker, Risharde Ramnath, Stephen Ficklin, Margaret Staton, Frank A Feltus, Sook Jung, Doreen Main, Jill L Wegrzyn

**Affiliations:** 1 Department of Ecology and Evolutionary Biology, University of Connecticut, Storrs, CT, USA; 2 Department of Horticulture, Washington State University, Pullman, WA, USA; 3 Department of Entomology and Plant Pathology, University of Tennessee, Knoxville, TN, USA; 4 Department of Genetics and Biochemistry, Clemson University, Clemson, SC, USA

The ‘Database URL’ has been changed to link to https://treegenesdb.org/Drupal

